# Therapeutic targeting of Krüppel-like factor 4 abrogates microglial activation

**DOI:** 10.1186/1742-2094-9-57

**Published:** 2012-03-19

**Authors:** Deepak Kumar Kaushik, Rupanjan Mukhopadhyay, Kanhaiya Lal Kumawat, Malvika Gupta, Anirban Basu

**Affiliations:** 1 National Brain Research Centre, Manesar, Haryana -122050, India

## Abstract

**Background:**

Neuroinflammation occurs as a result of microglial activation in response to invading micro-organisms or other inflammatory stimuli within the central nervous system. According to our earlier findings, Krüppel-like factor 4 (Klf4), a zinc finger transcription factor, is involved in microglial activation and subsequent release of proinflammatory cytokines, tumor necrosis factor alpha, macrophage chemoattractant protein-1 and interleukin-6 as well as proinflammatory enzymes, inducible nitric oxide synthase and cyclooxygenase-2 in lipopolysaccharide-treated microglial cells. Our current study focuses on finding the molecular mechanism of the anti-inflammatory activities of honokiol in lipopolysaccharide-treated microglia with emphasis on the regulation of Klf4.

**Methods:**

For *in vitro *studies, mouse microglial BV-2 cell lines as well as primary microglia were treated with 500 ng/mL lipopolysaccharide as well as 1 μM and 10 μM of honokiol. We cloned full-length Klf4 cDNA in pcDNA3.1 expression vector and transfected BV-2 cells with this construct using lipofectamine for overexpression studies. For *in vivo *studies, brain tissues were isolated from BALB/c mice treated with 5 mg/kg body weight of lipopolysaccharide either with or without 2.5 or 5 mg/kg body weight of honokiol. Expression of Klf4, cyclooxygenase-2, inducible nitric oxide synthase and phospho-nuclear factor-kappa B was measured using immunoblotting. We also measured the levels of cytokines, reactive oxygen species and nitric oxide in different conditions.

**Results:**

Our findings suggest that honokiol can substantially downregulate the production of proinflammatory cytokines and inflammatory enzymes in lipopolysaccharide-stimulated microglia. In addition, honokiol downregulates lipopolysaccharide-induced upregulation of both Klf4 and phospho-nuclear factor-kappa B in these cells. We also found that overexpression of Klf4 in BV-2 cells suppresses the anti-inflammatory action of honokiol.

**Conclusions:**

Honokiol potentially reduces inflammation in activated microglia in a Klf4-dependent manner.

## Introduction

Microglia, the resident macrophages of the central nervous system (CNS), are amongst the most important cells of innate immunity within this organization and become activated in response to pathological stimuli, such as endotoxins and other insults to the CNS [1,2]. Activated microglia respond by secreting proinflammatory cyto-chemokines such as macrophage chemoattractant protein-1 (MCP-1), TNF-α and IL-6 as well as proinflammatory enzymes such as inducible nitric oxide synthase (iNOS) and cyclooxygenase-2 (Cox-2) [3-6]. These exaggerated responses by microglia can result in neuronal damage [7] that can be detrimental to normal CNS functions [1]. Microglial inflammation is also observed in several neurodegenerative diseases including Alzheimer's disease [8], Parkinson's disease and multiple sclerosis [9,10]. Understanding the molecular mechanisms of microglial activation and targeting the key players of inflammation within microglia are essential in designing therapeutic strategies to deal with inflammatory conditions of the CNS.

Phospho-nuclear factor-κB (pNF-κB) is one of the most studied transcription factors for inflammatory pathways and is attributed with the upregulation of these proinflammatory cytokines [5,6]. In addition to pNF-κB, zinc finger transcription factor Krüppel-like factor 4 (Klf4) has been reported to play a key role in endothelial- and macrophage-mediated inflammation [11-16] alongside its role in cellular growth and differentiation in the epithelial lining of the gut and skin [17,18]. More recently, our laboratory has shown that Klf4 is important in mediating the upregulation of these proinflammatory cytokines in microglial cells [19]. We observed a significant increase in the expression of total as well as nuclear Klf4 in microglial cells upon lipopolysaccharide (LPS) treatment in a time-dependent manner. Upon LPS treatment to BV-2 cells, Klf4 also interacted with the iNOS promoter, which was confirmed by electrophoretic mobility shift assay studies. During this study, we also observed that siRNA-mediated knockdown of Klf4 resulted in a significant reduction in the levels of these cytokines and proinflammatory enzymes. In addition, we found that Klf4 potentially interacted with pNF-κB in response to LPS, suggesting that the two transcription factors may be equally important in regulating the downstream proinflammatory markers of microglial activation. It is quite likely that the proinflammatory actions of Klf4 are associated with its interaction with pNF-κB. Since most studies have focused on the role played by pNF-κB in mediating inflammation, our main focus was to evaluate the inflammatory potential of Klf4. Our previous studies confirmed that Klf4 is a key transcription factor in modulating inflammation. We therefore wanted to investigate whether Klf4 can be a potential target for anti-inflammatory agents such as honokiol (HNK).

HNK is a biphenolic anti-inflammatory agent isolated from the barks of stems and roots of various *Magnolia *species and used as a traditional herbal medicine [20]. HNK possesses neuroprotective [21], anti-cancerous [22,23] and anti-inflammatory properties [24,25]. There is plenty of literature about the anti-inflammatory roles of HNK in dendritic cells [26], macrophages [27] and several other cellular systems [28-32]. It is reported that the anti-inflammatory actions of HNK in LPS-mediated inflammation act via p38, extracellular signal-regulated kinases 1/2 (ERK1/2) and c-Jun N-terminal kinases 1/2 (JNK1/2) pathways [27]. Recently, a study focusing on the use of HNK on gliosarcoma revealed that it can easily cross the blood-brain barrier and blood-cerebrospinal fluid barrier [22], which makes it a drug of choice for CNS disorders. Moreover, HNK does not antagonize the binding of LPS to its receptors and does not alter the surface expression of CD14 or toll-like receptor 4 [27]. In addition, HNK also inhibits cytotoxicity induced by LPS in RAW264.7 macrophage cells [25]. Whether HNK treatment modulates Klf4 expression upon LPS treatment and decreases microglial activation stimulation has never been studied. Therefore, this study focuses on understanding the role of HNK in modulating the expression of Klf4 as well as LPS-induced neuroinflammation. Our current study shows that HNK downregulates the expression of Klf4 and pNF-κB and suppresses the overall expression of iNOS and Cox-2 as well as MCP-1, IL-6 and TNF-α in LPS-treated microglia as well as in mouse brain. Our study highlights Klf4 as a potential target for therapeutic activities of HNK.

## Materials and methods

### Cells and culture reagents

Mouse microglial cell line BV-2 was originally obtained from Dr Steve Levison, University of Medicine and Dentistry, New Jersey, USA and maintained in the laboratory as previously described [19]. Briefly, BV-2 cells were grown at 37°C in DMEM supplemented with 5% sodium bicarbonate (NaHCO_3_), 10% FBS, penicillin at 100 units/mL and streptomycin at 100 μg/mL. All reagents related to cell culture were obtained from Sigma Aldrich, St. Louis, MO, USA unless otherwise stated. Primary mixed glial cultures were prepared from postnatal day 0-2 (P0-P2) mouse brains as described previously [19]. The cells were plated into 75-cm^2 ^tissue culture flasks at a density of 2 × 10^5 ^viable cells/cm^2 ^in minimum essential medium supplemented with 10% FBS, penicillin at 100 units/mL, streptomycin at 100 μg/mL, 0.6% glucose and 2 mM glutamine. Media were changed every two to three days after plating. On day 12, when the mixed glial culture was confluent, the flasks were shaken on an Excella E25 orbital shaker (New Brunswick Scientific, Edison, NJ, USA) at 250 rpm for 60 to 75 minutes to dislodge microglial cells which were then allowed to adhere in bacteriological petri dishes for 60 to 90 minutes. The adherent cells were scraped, centrifuged and plated in chamber slides at a density of 8 × 10^4 ^viable cells/cm^2^, and incubated at 37°C until the treatment.

### Lipopolysaccharide and honokiol treatment

For *in vitro *experiments, treatment involved a pre-incubation with HNK (dissolved in 1X PBS and dimethyl sulfoxide (DMSO) in a 1:1 ratio; Sigma Aldrich) for 3 hours in serum-free media and then co-treatment with LPS (Sigma Aldrich) for an additional 6 hours for reactive oxygen species (ROS) and nitric oxide (NO) generation for a luciferase assay, or for 12 hours for immunoblotting, immunocytochemistry and cytokine bead array (CBA) analysis, under serum-free conditions. The control cells received only serum-free culture media and a mixture of DMSO and 1X PBS in a 1:1 ratio at the same volume as HNK.

For *in vivo *experiments, three groups of 6- to 8-week-old BALB/c mice were injected intraperitoneally with 2.5 or 5 mg/kg body weight of HNK dissolved in 1X PBS and DMSO in a 1:1 ratio over 12 hours while the control group received the sham mixture (1X PBS and DMSO in a 1:1 ratio). After 12 hours of HNK administration, mice were injected intraperitoneally with 5 mg/kg body weight of *Salmonella enterica *LPS dissolved in 1X PBS for an additional 24 hours. Control animals received the same volume of 1X PBS alone. All the animals were killed after 24 hours of treatment for tissue or protein. All experiments were performed according to the protocol approved by the Institutional Animal Ethics Committee.

### Measurement of reactive oxygen species and nitric oxide

We examined the effects of LPS and HNK + LPS on microglial cells by determining the levels of ROS as well as NO. Intracellular ROS generation in control and treated cells was assessed using the cell permeable, non-polar hydrogen peroxide-sensitive dye 5-(and-6)-chlromethyl-2', 7'- dichlorodihydrofluorescein diacetate (Sigma Aldrich) and the mean fluorescent intensities (MFIs) were measured on the FL-1 channel on a fluorescence-activated cell sorting (FACS) Calibur flow cytometer (Becton Dickinson, Franklin Lakes, NJ, USA) as described previously [33]. For NO production, nitrite generation by BV-2 cells was used as an indicator of NO release and measured by the Griess reaction as described previously [19]. The readings were taken at 540 nm using a microplate spectrophotometer (Bio-Rad, Gladesville, NSW, Australia) and the concentration was calculated from a sodium nitrite standard reference curve.

### Immunoblotting

For *in vitro *studies, untreated control and treated BV-2 cells were lysed in buffer containing 1% Triton-X-100, 10 mM Tris(hydroxymethyl)aminomethane-Cl (pH 8.0), 150 mM sodium chloride, 0.5% octylphenoxypolyethoxyethanol (Nonidet P-40), 1 mM ethylenediaminetetraacetic acid, 0.2% ethylene glycol tetraacetic acid, 0.2% sodium orthovanadate and protease inhibitor cocktail (Sigma Aldrich).

For *in vivo *studies, the whole brain tissues, barring the olfactory lobes and the cerebellum, from three animals after 24 hours of LPS administration and untreated mock controls were dissected and homogenized in 1X PBS with protease inhibitor. Protein concentrations were determined using the bicinchoninic assay method and 30 μg of each protein sample was electrophoresed on a 7.5% to 10% SDS-PAGE and transferred onto a nitrocellulose membrane. After blocking in 5% skimmed milk in 1X PBS-Tween-20 (1X PBST) for 4 hours at room temperature, the blots were incubated in 1X PBST overnight at 4°C with gentle agitation with either rabbit anti-Klf4 (1:500; Chemicon International, Temicula, CA, USA), anti-iNOS (1:1,000; Millipore, Billerica, MA, USA), Cox-2 (1:1,000; Millipore), pNF-κB (1: 1,000; Ser-536; Cell Signaling Technology, Danvers, MA, USA) and anti-β-actin (1:10,000; Sigma Aldrich). After five washes in 1X PBST, blots were then incubated with goat anti-rabbit horseradish peroxidase (1:5,000; Vector Laboratories, Burlingame, CA, USA) for 1.5 hours, with agitation. The blots were developed by using chemiluminescence reagent (Millipore) in ChemiGenius Bioimaging System (Syngene, Cambridge, UK). The images were captured and analyzed using the GeneSnap and GeneTools software, respectively, from Syngene. The protein levels were normalized to β-actin levels.

### Luciferase assay

Luciferase reporter gene constructs pGL2-iNOS (kindly provided by Dr Mark A. Feinberg and Dr Mukesh K. Jain, Harvard Medical School, Boston, MA, USA; the construct was originally made by Dr Mark A. Perella, also Harvard Medical School) [12,34] and pCOX301 (a kind gift from Dr Manikuntala Kundu, Bose Institute, Kolkata, India) [35] were used, containing mouse iNOS promoter region (-1485 to +31 bp) and Cox-2 promoter region (-891 to +9 bp) cloned upstream of the luciferase reporter gene in the pGL2-promoter and pGL2-basic vector, respectively (Promega, Madison, WI, USA). The luciferase assay was carried out using the luciferase assay kit (Promega) according to the manufacturer's protocol and as described previously [19]. The reading was taken using a Sirius single tube luminometer (Berthold Detection Systems GmBH, Bad Wildbad, Germany). The luciferase units were measured as relative luciferase units and these values were normalized to the amount of protein present in the sample.

### Generation of Klf4 construct

Oligonucleotide primers were designed to amplify the coding sequence of full-length mouse Klf4 cDNA yielding a 1452 bp (1.4 kb) product. The oligonucleotide primers were as follows: forward primer: 5'-ATGAGGCAGCCACCTGGCGA-3'; reverse primer: 5'- TTAAAAGTGCCTCTTCATGTGTAA-3'. The amplified product was then electrophoresed onto 0.8% agarose and the 1452 bp fragment was gel extracted using a Gel Extraction Kit (Qiagen, Hilden, Germany). The fragment was then inserted into the pcDNA3.1/V5 His TopoTA expression vector (Invitrogen, Carlsbad, CA, USA) to generate a pcDNA3.1-Klf4 (K-10) construct which was sequenced commercially (ILS Bioservices, Manesar, India).

### Immunochemical studies

For immunocytochemical studies, mouse primary microglial cells were fixed in 4% paraformaldehyde for 20 minutes at 25°C post-treatment and then incubated in blocking solution for 1 hour at 25°C. The cells were then incubated with rabbit anti-Klf4 (1:250; Millipore) overnight at 4°C. Anti-rabbit Alexa fluor 594 secondary antibody (1:1,000; Vector Laboratories) was used as the secondary antibody and mounting was carried out with mounting medium containing 4,6-diamidino-2-phenylindole (DAPI; Vector Laboratories). Images were captured using a Zeiss Apotome microscope (20× magnification; Zeiss, Hamburg, Germany). For immunohistochemistry, LPS-treated and age-matched control BALB/c mice in sets of three were perfused transcardially with 1X PBS and isolated whole brains were fixed with 4% paraformaldehyde in 1X PBS for 24 hours at 4°C. These were then kept in 30% sucrose for about 24 to 48 hours at 4°C or until the tissue was immersed at the bottom of the tube. The brains were then sectioned on a cryostat and stained for a marker of activated microglia, Iba1, as described previously [19]. Briefly, microglia were labeled with rabbit anti-Iba1 antibody (1:250; Wako, Osaka, Japan) by incubating the sections overnight at 4°C. After five washes with 1X PBS, the sections were incubated with horse anti-mouse fluorescein isothiocyanate (1:250; Vector Laboratories) for 1.5 hours. The slides were then mounted with mounting medium containing DAPI and images were captured using a Zeiss Apotome microscope (40× magnification; Zeiss).

### Cytokine bead array

The CBA kit (BD Biosciences, Franklin Lakes, NJ, USA) was used for the quantitative measurement of cytokine levels in control, LPS and LPS + HNK conditions. Using 50 μL of mouse inflammation standard and sample dilutions, the assay was performed according to the manufacturer's instructions and analyzed on the FACSCalibur flow cytometer (Becton Dickinson). Analysis was performed using CBA software that allows the calculation of cytokine concentrations in unknown lysates as described previously [19,36]. The levels of cytokines were measured in picograms per milliliter.

### Statistical analysis

Data are represented as the mean ± standard error of the mean (SEM) from at least three independent experiments. The data generated were analyzed statistically by paired two-tailed Student's *t*-test. *P *< 0.01 and < 0.05 were considered significant.

## Results

### Honokiol reduces microglial activation *in vitro*

Treatment of BV-2 cells with LPS results in microglial activation that can be measured by the increase in the levels of the proinflammatory enzymes iNOS and Cox-2 as well as the proinflammatory cyto-chemokines, MCP-1, IL-6 and TNF-α [19]. TNF-α, an acute phase protein that is secreted by macrophages and microglia in response to pathogenic stimuli, MCP-1, known to recruit inflammatory cells into CNS parenchyma [37] and IL-6, which has pleotropic functions in the CNS [38,39], are all cytokines known to increase dramatically in response to inflammation and CNS injury. We observed a significant increase in the levels of these proinflammatory markers after 12 hours of LPS stimulation. For HNK work, we estimated the cell viability using different doses of HNK (data not shown) and found that 1 μM and 10 μM doses of HNK are non-toxic doses for *in vitro *work. We therefore used these doses for our *in vitro *experiments. We found that in LPS + HNK conditions, the levels of these cytokines are significantly reduced as compared to the LPS alone condition. The levels of iNOS were significantly reduced by more than two-fold in both 1 μM and 10 μM HNK conditions when compared with LPS alone (1A). However, there was no dose-dependent decrease in iNOS levels as the levels of iNOS upon treatment with both 1 μM and 10 μM HNK was not significantly different from each other. There was no significant reduction in the levels of Cox-2 upon treatment with 1 μM HNK but treatment with 10 μM HNK significantly reduced the Cox-2 level in BV-2 cells by 1.5-fold with respect to the LPS-treated control (1B). We then wanted to see the effect of HNK on the levels of LPS-induced proinflammatory cytokines in BV-2 microglial cells. For this study, we carried out CBA analysis, which revealed a marked reduction in the levels of MCP-1 (1C), IL-6 (1D) and TNF-α (1E) cyto-chemokines with respect to LPS-treated conditions. These observations show for the first time that HNK has anti-inflammatory properties in activated microglial cells *in vitro*. These findings support existing literature on the anti-inflammatory properties of HNK for peripheral inflammation.

**Figure 1:**
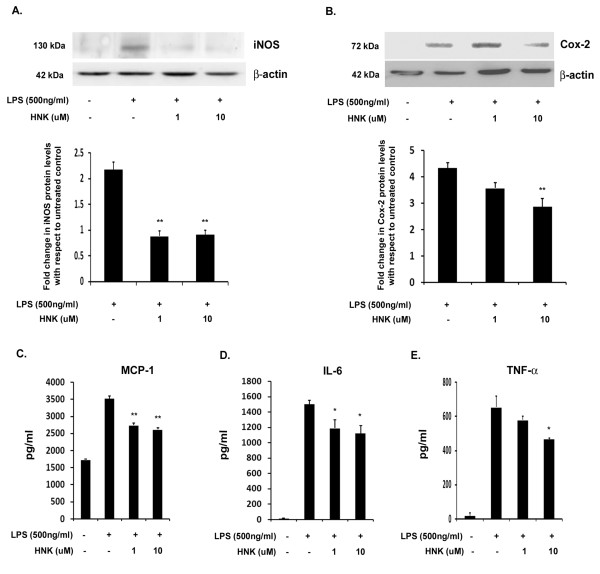
**Effect of honokiol on lipopolysaccharide-stimulated BV-2 cells**. Total cellular extract isolated from cells treated with 500 ng/mL of LPS and different doses of HNK for 12 hours were analyzed by immunoblotting. **(A) **Significant decrease in iNOS levels upon treatment with 1 μM and 10 μM HNK in LPS-treated cells with respect to LPS alone-treated cells. **(B) **Significant decrease in the expression of Cox-2 upon treatment with 10 μM HNK. The graphs represent fold change in protein levels relative to untreated controls. **(C-E) **CBA analysis of protein extract from BV-2 cells treated with LPS as well as in LPS + HNK conditions. CBA revealed a significant decrease in BV-2 cells treated with different doses of HNK in the levels of MCP-1 **(**C), IL-6 (D) and TNF-α (E) with respect to LPS. Absolute values of cytokines are represented in pg/mL. Data represent mean ± SEM from three independent experiments performed in duplicate. *P < 0.05 and **P < 0.01 in comparison to LPS-treated values.

### Honokiol decreases reactive oxygen species and nitric oxide generation in response to lipopolysaccharide treatment *in vitro*

Generation of ROS and NO by microglial cells in response to LPS is a key marker for oxidative stress in these cells. Earlier work has shown the anti-oxidant properties of HNK in monocytes and macrophages [25]. We therefore measured ROS and NO levels in LPS-treated microglial cells upon HNK treatment. Our findings suggest that HNK significantly reduces the production of both of these stress molecules. While LPS increased the formation of ROS by more than two-fold within 6 hours of stimulation (2A) as analyzed by measuring the MFIs using FACS, we observed that there was a significant reduction in the levels of ROS upon HNK treatment in a dose-dependent manner. While there was a reduction by 1.5-fold in ROS levels upon treatment with 1 μM HNK (2B), there was a more than two-fold reduction with 10 μM HNK (2C), thereby maintaining the ROS to the control levels (2D).

**Figure 2:**
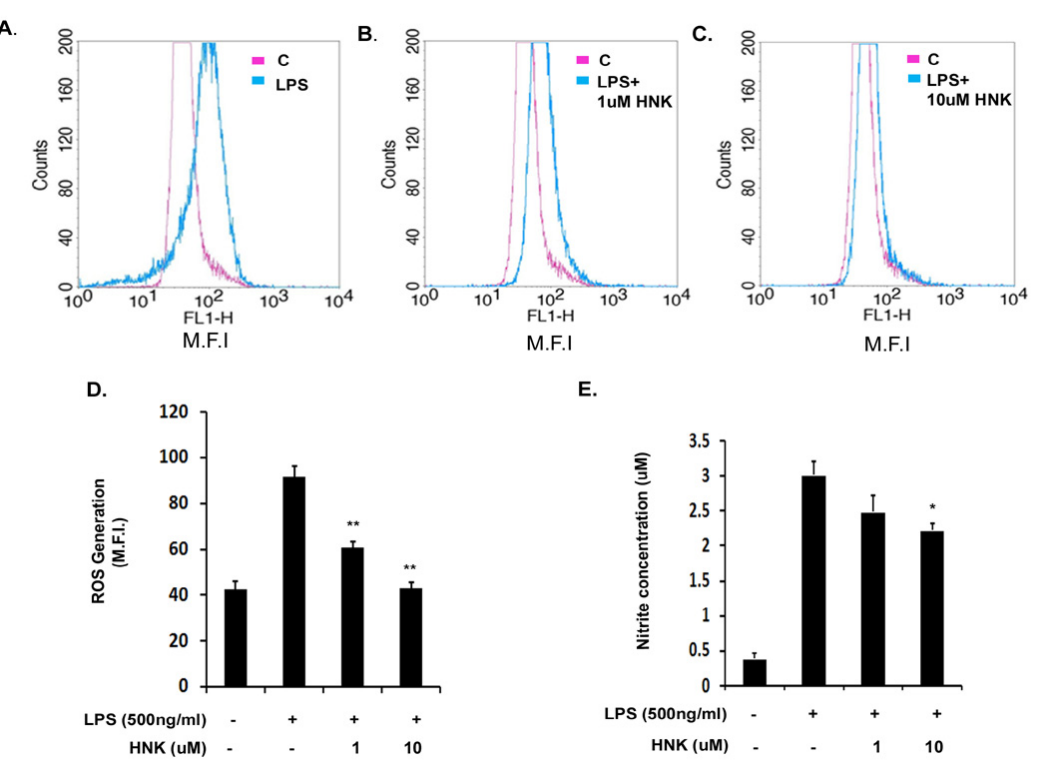
**Effect of honokiol on reactive oxygen species and nitric oxide generation in lipopolysaccharide-stimulated BV-2 cells**. Total cellular extract and supernatant detected for the levels of ROS and NO in LPS- and HNK-treated cells. Representative FACS plot showing intracellular ROS production. **(A) **Increase in the levels of ROS generation upon LPS treatment with respect to untreated control condition. **(B, C) **Decrease in ROS generation upon treatment with 1 μM and 10 μM HNK in LPS-stimulated cells. **(D) **Graph representing the MFI of ROS generation. **(E) **Graph representing nitrite concentration in LPS and LPS + HNK conditions. Nitrite is measured in μM quantities. Data represent mean ± SEM from three independent experiments performed in duplicate. *P < 0.05 and **P < 0.01 in comparison to LPS-treated values.

We also estimated nitrite production in order to measure NO release by LPS-stimulated BV-2 cells upon HNK treatment. Similar to our observations with ROS production, LPS increased generation of nitrite by more than 2.5-fold in comparison to the untreated control condition (2E), which was reduced by HNK treatment. Although the reduction was not significant with 1 μM HNK dose, we observed a significant decrease in NO levels with 10 μM HNK dose when compared with the LPS alone-treated condition (2E). These findings confirm that HNK reduces the oxidative stress generated in BV-2 microglial cells upon LPS stimulation. Reduction in NO production also supports our observation of a decrease in iNOS levels upon HNK treatment to LPS-stimulated cells.

### Honokiol suppresses the production of cyclooxygenase-2 and proinflammatory cyto-chemokines *in vivo*

From our findings so far we know that HNK reduces LPS-mediated inflammation *in vitro *where it is shown to suppress the production of proinflammatory enzymes as well as cyto-chemokines. In order to confirm whether HNK can reduce inflammation *in vivo *in mouse brain as well, we measured the levels of proinflammatory cytokines and Cox-2 in LPS-treated mice. While the levels of all these inflammatory markers were significantly elevated in mice administered with LPS alone, mice that were treated with HNK in addition to LPS showed a significant 1.5-fold reduction in the levels of Cox-2 (3A). When we analyzed the brain homogenate for cytokines, we found that, in LPS-treated mice, the levels of all these cytokines were elevated significantly with respect to control-treated mice. A dosage of 5 mg/kg body weight of HNK significantly reduced the levels of MCP-1 by about two-fold in mice (3B). Both the doses of HNK reduced the production of IL-6 by more than 1.5-fold (3C) and TNF-α by more than 1.4-fold (3D) with respect to the LPS alone-treated mouse group. These findings suggest that, in addition to its anti-inflammatory role *in vitro*, HNK can reduce inflammation of the CNS.

**Figure 3:**
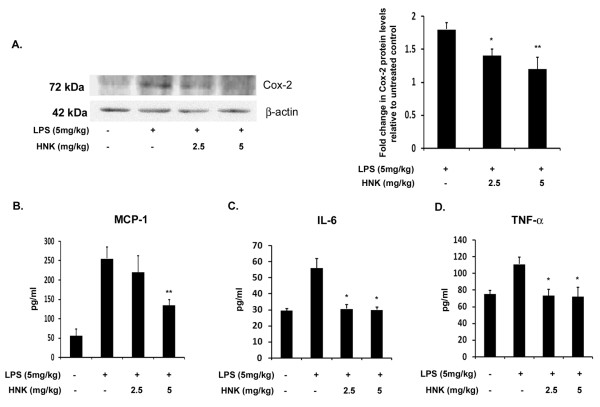
**Levels of cyclooxygenase-2 and proinflammatory cytokines in lipopolysaccharide- and honokiol-treated BALB/c mice**. BALB/c mice administered with different doses of HNK and 5 mg/kg body weight of LPS were analyzed by Cox-2 immunoblotting and CBA analysis. **(A) **Decrease in the levels of Cox-2 in mice treated with different doses of honokiol. Graph represents fold change in Cox-2 levels relative to untreated control condition. **(B-D) **CBA analysis of brain homogenates from BALB/c mice in different conditions. There was a significant decrease in the levels of MCP-1 (B), IL-6 (C) and TNF-α (D) in mice treated with different doses of HNK. Absolute values of cytokines are represented in pg/mL. Data represent mean ± SEM of five animals in each group. *P < 0.05 and **P < 0.01 in comparison to LPS-treated values.

### Honokiol reduces the promoter activity of inducible nitric oxide synthase and cyclooxygenase-2

It is evident from our studies so far that HNK downregulates the production of iNOS and Cox-2. In order to confirm whether HNK reduces the promoter activities of iNOS and Cox-2, we carried out promoter binding assays using pGL2-iNOS and pCOX301 constructs. We found that while LPS treatment enhanced the promoter activities of iNOS and Cox-2 by three- and two-fold respectively, HNK treatment significantly reduced the promoter activities of both iNOS and Cox-2 (4A, B). While 1 μM HNK was enough to bring down the iNOS promoter activity, Cox-2 promoter binding activity was severely affected with 10 μM HNK after 6 hours of LPS stimulation. The reduction in promoter activities of iNOS and Cox-2 upon HNK treatment suggests that HNK inhibits the binding of transcription factors to their respective promoters in BV-2 microglial cells.

**Figure 4:**
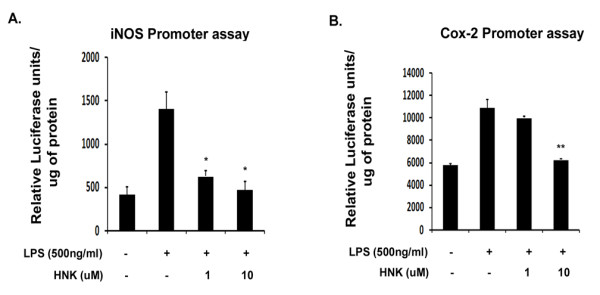
**Inducible nitric oxide synthase and cyclooxygenase-2 promoter assay in response to lipopolysaccharide and honokiol**. Luciferase assay from total protein extracts from pGL2-iNOS and pCOX301 transfected, LPS- and HNK-treated BV-2 cells. **(A) **Decrease in iNOS promoter activities upon stimulation with different doses of HNK with respect to LPS-treated condition. **(B) **Decrease in Cox-2 promoter activities upon stimulation with 10 μM HNK in comparison to LPS-treated condition. Graphs represent relative luciferase units normalized to protein levels. Data represent mean ± SEM from three independent experiments performed in duplicate. *P < 0.05 and **P < 0.01 in comparison to LPS-treated values.

### Honokiol reduces the expression of Krüppel-like factor 4 and phospho-NF-κB *in vitro*

LPS induces the expression of pNF-κB in macrophages as well as in microglial cells. We have previously shown that LPS induces both the expression of pNF-κB as well as Klf4 in BV-2 cells and primary microglia [19]. We wanted to see if HNK downregulates the expression of these two transcription factors in microglia upon activation. We found that HNK significantly reduces the expression of both pNF-κB and Klf4 in a dose-dependent manner in BV-2 cells. We have observed a reduction of 1.4-fold in pNF-κB levels with 1 μM HNK while there was about two-fold reduction in its levels when LPS-treated cells were co-treated with 10 μM HNK (5A) with respect to LPS alone-treated cells. We found similar results when we looked at the levels of Klf4. Significantly, there was a more than two-fold reduction in the levels of Klf4 with both 1 μM and 10 μM HNK doses (5B) with respect to LPS-treated cells. To the best of our knowledge, this is the first report showing that Klf4 can be downregulated by any anti-inflammatory agent. The reduction in Klf4 and pNF-κB levels confirm HNK's anti-inflammatory properties in microglial activation as both these transcription factors are associated with proinflammatory activities in these cells.

**Figure 5:**
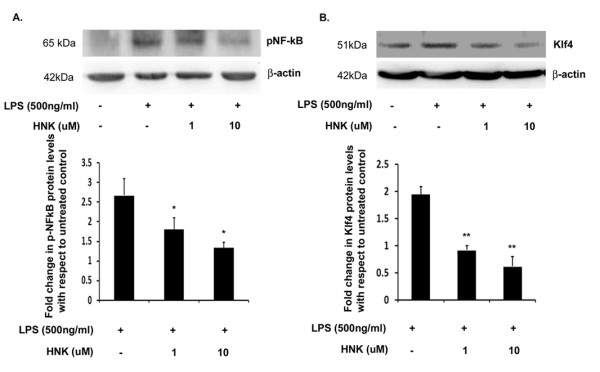
**Effect of honokiol on the expression levels of phospho-NF-κB and Krüppel-like factor 4 in BV-2 cells**. Total cellular extract isolated from cells treated with 500 ng/mL of LPS and different doses of HNK for 12 hours were analyzed by immunoblotting for Klf4 and pNF-κB levels. **(A) **Decrease in pNF-κB levels upon treatment with different doses of HNK. **(B) **Significant decrease in Klf4 levels upon treatment with different doses of HNK. The graphs represent fold change in protein levels relative to untreated controls. Data represent mean ± SEM from three independent experiments performed in duplicate. *P < 0.05 and **P < 0.01 in comparison to LPS-treated values.

### Honokiol reduces the production of Krüppel-like factor 4 and phospho-NF-κB *in vivo*

To corroborate our *in vitro *findings with regards to the regulation of Klf4 and pNF-κB, we estimated the levels of these two transcription factors in mouse brain upon treatment with HNK and LPS. Similar to our observations with BV-2 cells, we found that there was an overall reduction in the levels of both pNF-κB and Klf4 in HNK-treated mice when compared to the LPS alone-treated group. There was a more than 1.5-fold decrease in the levels of both of these transcription factors when mice were treated with 2.5 and 5 mg/kg body weight of HNK (6A, B) with respect to LPS-treated mice. The reduction in the levels of these transcription factors is crucial for controlling inflammation which is induced by LPS in the brain.

**Figure 6:**
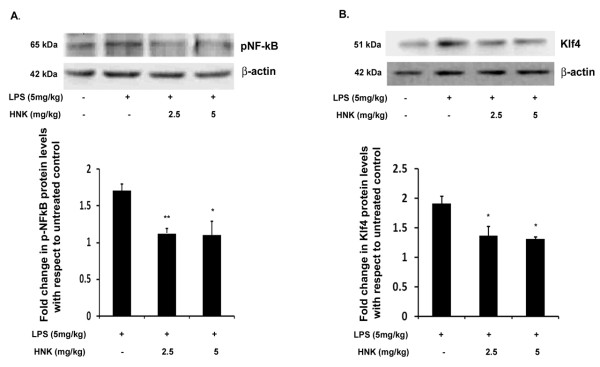
**Effect of honokiol on the expression levels of phospho-NF-κB and Krüppel-like factor 4 *in vivo***. Brain homogenate from BALB/c mice treated with different doses of HNK and 5 mg/kg body weight of LPS analyzed by immunoblotting for Klf4 and pNF-κB levels. **(A) **Significant decrease in the levels of pNF-κB in 2.5 as well as 5 mg/kg body weight dose of HNK with respect to LPS-treated mice. **(B) **Significant decrease in Klf4 levels in 2.5 as well as 5 mg/kg body weight HNK doses with respect to LPS-control mice. Graphs represent fold change in protein levels with respect to untreated control condition. Data represent mean ± SEM of five animals in each group. *P < 0.05 and **P < 0.01 in comparison to LPS-treated values.

### Honokiol reduces Krüppel-like factor 4 expression and activation of primary microglia

While we have confirmed that Klf4 expression in response to LPS is suppressed by treatment with HNK *in vitro *in BV-2 cells as well as *in vivo *in BALB/c mice, we wanted to study whether HNK induces any such changes in primary microglia. Immunocytochemical studies reveal that treatment with LPS for 12 hours increased Klf4 expression in primary microglia (7B) with respect to untreated controls (7A). In LPS-treated primary microglia, we observed elongated microglial processes within 12 hours of LPS treatment, suggesting an activated morphology. However, upon treatment with 10 μM HNK, we saw a reduction in Klf4 expression in primary microglia cells and the processes that were extended upon LPS exposure were markedly reduced (7C). The effect of HNK on the activation of primary microglia and Klf4 expression supports our previous observations of HNK's effect in culture as well as in mice models.

**Figure 7:**
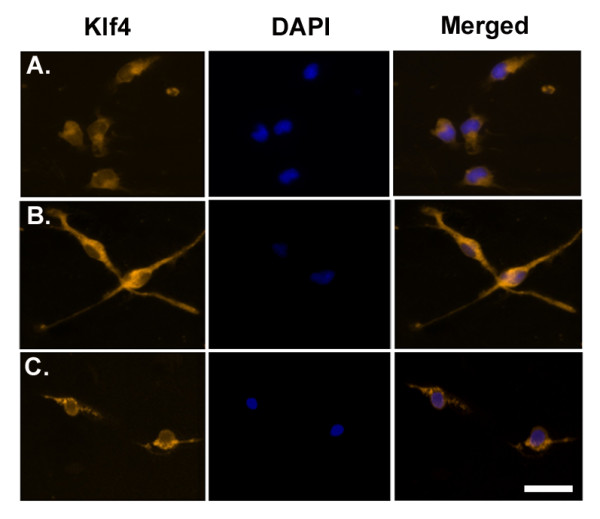
**Effects of honokiol on Krüppel-like factor 4 expression in lipopolysaccharide-treated primary microglia**. Primary microglia isolated from P0-P2 BALB/c mice treated with 500 ng/mL of LPS and 10 μM of HNK were analyzed by immunocytochemistry. **(A) **Fluorescent images of untreated primary microglia treated with a sham mixture of 1X PBS and DMSO in a 1:1 ratio. **(B) **Primary microglia treated with LPS for 12 hours and **(C) **LPS-treated primary microglia co-treated with HNK for 12 hours. Images showing the staining for Klf4 (Alexa fluor 594; Rhodamine channel); DAPI (blue) and merged images (right panel). Scale bar: 20 μm.

### Honokiol reduces microglial activation in mouse cortex

So far, it is clear from our findings that HNK reduces inflammation in the brain. Whether HNK brings down microglial activation in the mouse brain was confirmed by immunohistochemical analysis of the mouse brain cortex. The group that received LPS alone showed a marked activation of microglia as shown by an arrow in 8B compared to the untreated control group (8A) after 24 hours of LPS treatment. These activated microglia show a bushy appearance and have a large number of activated processes. However, the images from the mouse brain receiving HNK suggest a marked reduction in activation of microglia (8C, D). Upon treatment with 2.5 and 5 mg/kg body weight of HNK, the microglia exhibited a 'resting' morphology similar to that observed in a control mouse brain. These findings show that these doses of HNK are effective in reducing microglial activation *in vivo*, thereby reducing inflammation in the mouse brain.

**Figure 8:**
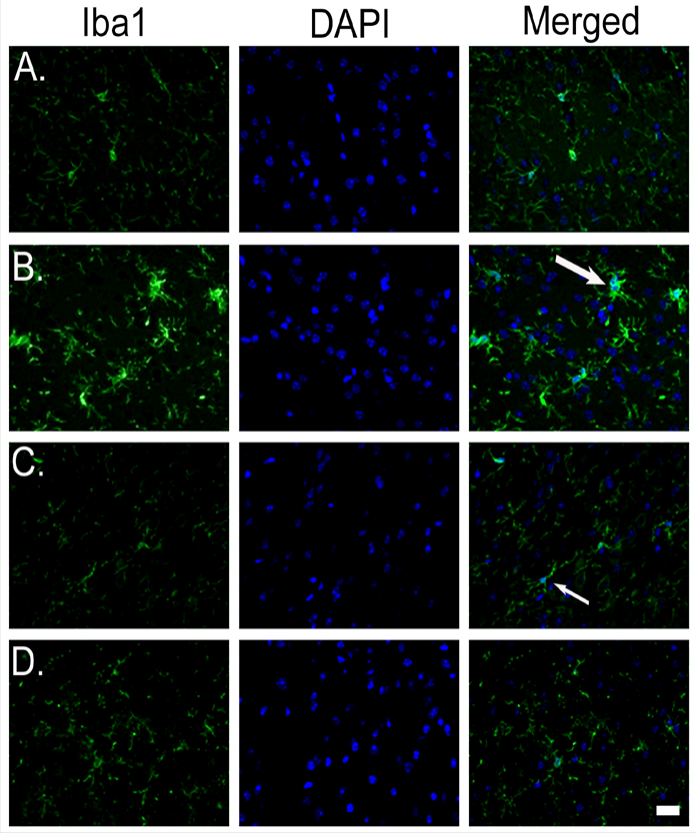
**Honokiol-mediated reduction in microglial activation *in vivo***. Cryostat sections from BALB/c mice treated with different doses of HNK and 5 mg/kg body weight of LPS, analyzed by immunohistochemistry. **(A) **Fluorescent images of brain section from control mice showing microglia in their 'resting' morphology. **(B) **Images showing the an increase in the number of 'activated' microglia in response to LPS as shown by an arrow. **(C, D) **Images from mice treated with 2.5 mg/kg body weight of HNK (C) and 5 mg/kg body weight of HNK (D) showing microglia with resting stage morphology similar to the control condition, marked by a small arrow. Images show the staining for Iba-1 (fluorescein isothiocyanate, green), DAPI (blue) and merged images (right panel). Scale bar: 50 μm.

### Krüppel-like factor 4 overexpression increases inflammation and suppresses the anti-inflammatory activity of honokiol

According to our earlier reports, knockdown of Klf4 in LPS-treated BV-2 cells decreased inflammation [19]. To find out whether overexpression of Klf4 can increase inflammation in these cells and whether it can override HNK's anti-inflammatory action of downregulating the production of proinflammatory enzymes, we transfected BV-2 cells with pcDNA3.1-Klf4 construct (K-10) and measured the levels of Klf4, iNOS and Cox-2 in different conditions. The control cells either received only lipofectamine (untreated condition) or were transfected with pcDNA3.1-LacZ construct (LacZ) for transfection control. Upon LPS stimulation in K-10 transfected cells, there was a significant induction in the levels of Klf4 with respect to the LPS alone-treated condition (9A). Treatment with 10 μM HNK resulted in a significant reduction of Klf4 in K-10 + LPS cells. However, the levels of Klf4 were still significantly higher than those observed in the LPS alone condition. Similar observations were made when the levels of iNOS and Cox-2 were measured in these conditions. iNOS and Cox-2 levels increased significantly in K-10 + LPS cells with respect to the LPS alone condition, confirming our previous findings that Klf4 is involved in the upregulation of these proinflammatory enzymes (9B, C). In addition to these findings, we determined that treatment with 10 μM HNK did not significantly reduce Cox-2 or iNOS levels in K-10 + LPS cells, which remained significantly higher with respect to the LPS alone condition (9B, C). While it is clear from this study that Klf4 promotes inflammation, it is also noteworthy that Klf4 overexpression suppresses the anti-inflammatory action of HNK. We can infer from these findings that the targeting of Klf4 by HNK is a crucial event required for its anti-inflammatory properties.

**Figure 9:**
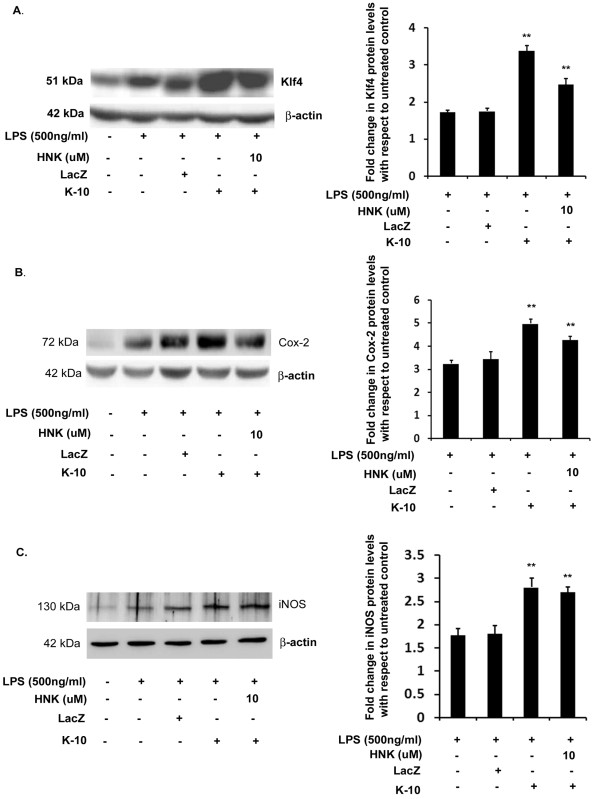
**Effect of Krüppel-like factor 4 overexpression on the anti-inflammatory properties of honokiol**. Total cellular extract isolated from cells transfected with K-10 and LacZ constructs and treated with 500 ng/mL of LPS along with different doses of HNK, analyzed by immunoblotting for Klf4, iNOS and Cox-2. **(A) **Expression levels of Klf4 in different conditions showing increased expression of Klf4 in LPS-treated with respect to untreated control as well as a significant increase in Klf4 in K-10 + LPS cells with respect to LPS alone condition. **(B) **Cox-2 expression showing increased expression under K-10 + LPS as well as HNK-treated K-10 + LPS conditions with respect to LPS alone or control untreated condition. **(C) **Expression levels of iNOS showing significant increase in its levels in K-10 + LPS and K-10 + LPS conditions treated with HNK compared to LPS alone-treated condition. Graphs represent fold change in protein levels with respect to untreated control condition. Data represent mean ± SEM from three independent experiments performed in duplicate. **P < 0.01 in comparison to LPS-treated values.

## Discussion

Inflammation of the CNS is marked by the excessive production of numerous proinflammatory cyto-chemokines including TNF-α, MCP-1 and IL-6 in response to LPS [3,4]. Previous reports from our laboratory have suggested that Klf4 is involved in mediating microglial activation and the regulation of proinflammatory mediators in response to LPS [19]. This study revealed HNK for the first time as an important factor regulating inflammation in microglial cells in addition to pNF-κB, another key player in inflammatory pathways. The focus of our study was to determine whether HNK, a biphenolic compound that can easily cross the blood-brain barrier [22] and also has a neuroprotective role in the brain [40], can reduce microglial activation and whether these activities are mediated by regulating Klf4. For our experiments, we induced inflammation in BV-2 cells and BALB/c mice using LPS and then treated them using HNK, whose anti-inflammatory role in the CNS has not previously been studied. Our studies show that HNK significantly reduces microglial activation and secretion of proinflammatory cyto-chemokines in response to LPS; that HNK downregulates both Klf4 and pNF-κB *in vitro *as well as *in vivo*; and that overexpression of Klf4 suppresses the anti-inflammatory properties of HNK.

**Figure 10:**
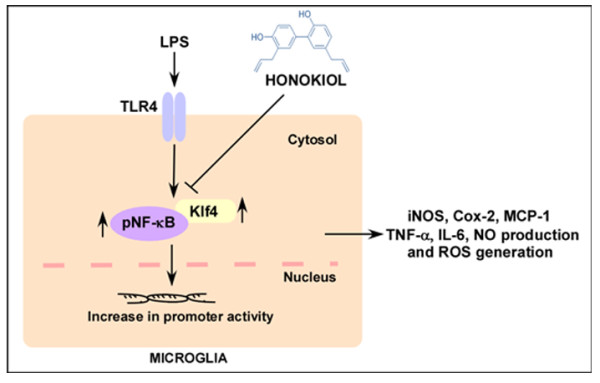
**Schematic showing the possible mode of action of honokiol**. LPS stimulation results in the overexpression of Klf4 and pNF-κB in microglial cells, which migrate to the nucleus and enhance the promoter activities of proinflammatory genes, thereby resulting in microglial activation. HNK inhibits the expression of these transcription factors as well as iNOS and Cox-2 promoter activities in LPS-stimulated microglial cells. HNK also suppresses the production of neuroinflammatory molecules, including TNF-α, MCP-1 and IL-6. In addition, HNK blocks NO production and ROS generation from activated microglia.

Endotoxins such as bacterial LPS can result in systemic inflammation and induce severe neuroinflammation, resulting in the production of iNOS and Cox-2 proinflammatory enzymes as well as other cytokines including TNF-α, MCP-1 and IL-6, which are potent mediators of inflammation in CNS [3,4]. This can be suppressed using anti-inflammatory agents which usually target pNF-κB pathways. iNOS catalyzes the production of NO, which can be neurotoxic at high concentrations [41,42]. On the other hand, Cox-2 catalyzes the production of prostaglandins such as prostaglandin E2 from arachidonic acid, which are then converted to active prostanoids by synthases [35,43]. The exaggerated production of these cytokines along with iNOS and Cox-2 can be deleterious to neuronal health. It is therefore necessary to find transcription factors which regulate their production. It is well known that, upon stimulation with LPS, pNF-κB binds to iNOS and Cox-2 promoter elements [6,44-46] and upregulates their production. We have previously shown that Klf4 also interacts with iNOS and Cox-2 promoter elements in response to LPS in microglial cells [19]. HNK can induce inflammation via different pathways, including p38, ERK1/2 and JNK1/2 as well as the phosphoinocytide-3 kinase/AKT (protein kinase/AKT) pathway [27,47], in addition to targeting pNF-κB [25,48]; we propose that HNK might also downregulate Klf4, thereby regulating iNOS and Cox-2 levels. It is quite likely that HNK, in addition to reducing pNF-κB and Klf4 expression, may inhibit the interactions between these two transcription factors upon LPS stimulation as was reported in our previous study [19]. However, further studies are required to study this activity of HNK in microglia. These inhibitory actions of HNK may result in the overall suppression of the proinflammatory state of microglia. Our present study shows that HNK not only inhibits Klf4 and downregulates the production of iNOS and Cox-2, it also dampens the production of other proinflammatory cyto-chemokines such as MCP-1, IL-6 and TNF-α as well as generation of NO in microglial cells *in vitro*. These properties of HNK are demonstrated in the schematic diagram representing its mode of action in LPS-treated microglia (Figure 10).

HNK also suppresses neuroinflammation stimulated by LPS in the mouse brain and induces the activation of microglia within the brain cortex of LPS-stimulated mice. Microglia in HNK + LPS mice resemble the resting state morphology, clearly showing that HNK does reduce neuroinflammation. It would be of interest to understand the role played by HNK in affecting other cell types of the brain as well as its underlying molecular mechanisms as other cell types, including astrocytes, also have been reported to have neuromodulating properties.

Oxidative stress is an important feature of inflammation and the role of HNK for its anti-oxidant properties have been studied in detail in the peripheral system and there is plenty of literature on its mode of action. Studies on collagen-induced arthritis in mice have shown that HNK suppresses production of TNF-α and IL-1β along with a reduction in oxidative damage [49]. Studies on ischemic injury in rats have shown that HNK can act as a potent ROS inhibitor by potentially modulating the enzymes in ROS production, such as myeloperoxidase, Nicotinamide adenine dinucleotide phosphate oxidase and Glutathione peroxidase in neutrophils [50,51]. In cerebellar granule neuronal cells, HNK was found to reverse hydrogen peroxide-mediated neurotoxicity [21]. Similarly, HNK administration resulted in a significant suppression of oxidative damage in mouse brain treated with N-methyl-d-aspartic acid [52]. In consensus with these studies, we report that HNK can significantly reduce the production of ROS in LPS-stimulated microglial cells. However, the detailed mechanisms of ROS inhibition by HNK in activated microglia is not clearly understood. It would be interesting to determine the mechanisms by which HNK mediates these anti-oxidant activities.

Our studies further identify a crucial role for Klf4 in mediating inflammation in BV-2 cells. We demonstrate that overexpression of Klf4 increases the production of iNOS and Cox-2 in these cells. In addition to these observations, we also report that Klf4 overexpression results in a decrease of anti-inflammatory activity of HNK. In K-10 + LPS conditions, HNK was not able to decrease the production of iNOS and Cox-2 significantly with respect to the K-10 + LPS alone condition. In addition, the levels of iNOS as well as Cox-2 remained significantly higher in the HNK treatment group with respect to the LPS alone condition. It is likely that overexpressed Klf4 remains bound to the respective promoter elements in spite of HNK treatment to these cells. There is, however, some reduction observed when HNK treatment was carried out in these Klf4 overexpressing cells which may be due to HNK's action on the basal level of cellular Klf4. These findings strongly suggest that Klf4 is one of the key players of inflammation and therefore one of the key targets of therapeutic intervention.

We hereby propose that Klf4 may be an important target for drugs that are known to traditionally block pNF-κB pathways. It would be interesting to understand the molecular mechanisms of HNK-mediated Klf4 expression. Furthermore, Klf4 as a potential target for other anti-inflammatory agents needs to be further evaluated.

## Abbreviations

Bp: base pair; CBA: cytokine bead array; CNS: central nervous system; Cox-2: cyclooxygenase-2; DAPI: 4,6-diamidino-2-phenylindole; DMEM: Dulbecco's modified Eagle's medium; DMSO: dimethyl sulfoxide; FACS: fluorescence-activated cell sorting; FBS: fetal bovine serum; HNK: honokiol; IL-6: interleukin-6; iNOS: inducible nitric oxide synthase; kb: kilobase; Klf4: Krüppel-like factor 4; K-10: pcDNA3.1-Klf4 construct; Lac-Z: pcDNA3.1-LacZ construct; LPS: lipopolysaccharide; MCP-1: macrophage chemoattractant protein-1; MFI: mean fluorescent intensity; NO: nitric oxide; PBS: phosphate-buffered saline; pNF-κB: phospho-nuclear factor kappa B; ROS: reactive oxygen species; SEM: standard error of the mean; siRNA: small interfering RNA; TNFα: tumor necrosis factor alpha; 1X PBST: 1X PBS-Tween-20.

## Competing interests

The authors declare that they have no competing interests.

## Authors' contributions

DKK designed and performed the experiments, analyzed the data and drafted the manuscript. RM, KK and MG performed the experiments. AB participated in the design and coordination of the experiments. All the authors reviewed the data and contributed to the preparation of the manuscript. All the authors have read and approved the final version of the manuscript.
